# Co-creative art processes with patients: A theoretical framework and qualitative study among artists

**DOI:** 10.1371/journal.pone.0266401

**Published:** 2022-04-07

**Authors:** Yvonne Weeseman, Michael Scherer-Rath, Nirav Christophe, Henny Dörr, Zarah M. Bood, Mirjam A. G. Sprangers, Esther Helmich, Hanneke W. M. van Laarhoven

**Affiliations:** 1 Department of Medical Oncology, Cancer Center Amsterdam, Amsterdam University Medical Centers, University of Amsterdam, Amsterdam, The Netherlands; 2 Faculty of Philosophy, Theology and Religious Studies, Radboud University, Nijmegen, The Netherlands; 3 HKU University of the Arts Utrecht, Utrecht, The Netherlands; 4 Department of Medical Psychology, Amsterdam Public Health, Amsterdam University Medical Centers, University of Amsterdam, Amsterdam, The Netherlands; 5 Amsta Healthcare Organization, Amsterdam, The Netherlands; The University of Hong Kong, HONG KONG

## Abstract

A cancer diagnosis may be experienced as a contingent life event. Co-creation—in which artists together with patients create a work of art reflecting on aspects of the patients’ life story—may be used to support patients to integrate such a contingent life event into their life story. We conducted a qualitative study in which we interviewed 10 professional artists to explore if co-creative art processes could facilitate integration of experiences of contingency in patients. Template analyses were performed in AtlasTi. We identified co-creation as a specific form of support to the process of integration of experiences of contingency. In the formation of a new life narrative, patients transcend the boundaries of their previous life narrative by changing their perspective. Self-transcendence forms a pivotal point in co-creation, which may be helpful for patients to integrate experiences of contingency into their life narratives.

## 1. Introduction

Current research efforts investigate the supportive effects of art, and more specifically co-creation, for patients with life threatening diseases such as cancer [1]. In co-creation artists together with patients create a work of art reflecting on aspects of the patients’ life story. Co-creation can be described as interactive engagement, which creates connection and trust, whereby process and product are both important and intertwined [1]. Artist and participant engage in a relationship where the artist has a leading role as initiator of the co-creation process while at the same time carefully shaping a high level of alignment [2]. Co-creation is based on shared ownership with equivalent contributions. Artists provide specific professional knowledge to facilitate the creational process and the other participant(s) provide(s) the content for co-creation [1]. Through the use of the senses and symbolic language co-creation may facilitate imagination and receptivity, enabling multiple ways of expression [1, 3, 4]. Co-creation may be regarded a ‘ritualizing process’ as both co-creation and rituals have the ability to channel anxiety evoked by a life event. They offer a means to encompass with reverence what in the reality of ordinary life might feel disturbing, unfamiliar, too complex, too simple, or too special to relate to [5, 6]. These processes evolve in liminal space, a transitional state where ‘one is not here anymore, and not yet there either’ [5, 6]. The work of art could then be seen as a ‘transactional object’ on which the patient projects an (emotional) meaning beyond the actual properties of the object [4]. Engaging in transactional objects could facilitate a movement towards self-transcendence, thus supporting one to take different viewpoints into account [7].

Following the above, co-creation could support a process of integration of life events into one’s life narrative. Yet, this has not been empirically investigated. First, in section 2, we will outline the theoretical framework in this study. Next, by interviewing professional artists, we aim to empirically investigate the concept of co-creation, and more specifically whether and—if so—how it supports a process of integration of life events into the life narrative.

## 2. Theoretical framework

### 2.1 Life events and experiences of contingency

Life events are generally described to be of a contingent nature as they are ‘neither impossible nor necessary, it could have happened otherwise’ [8]. Here, we further specify a contingent life event as a new, irreversible event introducing an undesired future, which is eminent and threatens one’s existence and life goals [9–11]. The diagnosis of cancer is an example of such a life event that could induce an experience of contingency. It can be perceived as a devastating disruption of one’s life narrative to the extent of an existential crisis: one starts to wonder and doubt the fundamentals of one’s own life [12–15].

### 2.2 Life narratives

Starting in early childhood, people develop internal representations and life narratives, to try to navigate their worlds, which in turn provides a sense of predictability, coherence and meaning [16–19]. An individual’s life narrative is not necessarily an attempt for a truthful reflection of reality or gaining an empirically based understanding of one’s own actions. It is merely an attempt to create a suitable reality [20]. Narratives are also used when life experiences deviate from what is common or expected. According to the philosopher Ricoeur, the process of constructing and reworking the personal narrative can be described as a process called ‘mimesis’.

### 2.3 Mimesis

One’s personal life narrative may be regarded an imitation of human action: ‘mimesis’. Reality is creatively interpreted in stories where stories and reality influence each other. Mimesis supports an understanding of the world, offers explanations and leads to a continuous process of (re)interpretation of one’s own (narrative) identity. The mimesis process consists of three iterative phases [21, 22].

The first phase, mimesis 1, or prefiguration, offers the already present elements and networks needed for the development of stories and one’s narrative identity including symbols, figures, actions, temporal and spatial structures. In mimesis 1 the person has inner knowledge of the blueprint of anticipated human action. Experiences are known within a timeframe of the past, present and future, but are not yet consciously structured into a narrative.

In the second phase, mimesis 2, or configuration, an ordered narrative is created by connecting events, persons and objects, endowing them with meaning within a larger whole, creating a sense of necessity, while at the same time contingency remains. Heterogeneous elements are brought together into a tentative state of concordant discordance, which means the events are still contingent and disrupting the life narrative, while at the same time being embedded within a larger whole of internal coherence, which serves to give these events a meaningful place.

In the third phase, mimesis 3, or refiguration, the ordered narrative from phase 2 is integrated into the previously existing life narrative, forming a new life narrative. The person is able to let go of the previous narrative, and can perceive the ‘new composition’ from a broader perspective. The new narrative is finally made one’s own.

Mimesis describes the human capacity to acknowledge contingency and integrate experiences of contingency into the life narrative [21–23].

### 2.4 Integration of experiences of contingency

Particularly in case of experiences of contingency, these experiences need to be made sense of and need to be integrated in the life narrative to restore coherence. The experience of contingency is reinterpreted in the context of one’s life narrative and new meanings might be attributed. In order to successfully integrate these experiences, the life narrative needs to be revised and include the ‘new normal’. The revision of the life narrative is an iterative process where the life narrative on specific events is being reworked and different viewpoints and new meanings are being explored [24, 25]. If reworking of an experience of contingency is blocked, it could become more difficult to allow new perspectives [26]. The integration of an experience of contingency may be facilitated by self-transcendence [10, 11, 27]. For this study self-transcendence is conceptualised as ‘a change in one’s consciousness where one is pulled beyond the boundaries of one’s self and is able to come to a different or new perspective on one’s life and can expand beyond one’s current life narrative’ [7]. Thus, self-transcendence facilitates the process of formation of a new life narrative.

### 2.5 Synthesis of the theoretical framework

**Fig 1:**
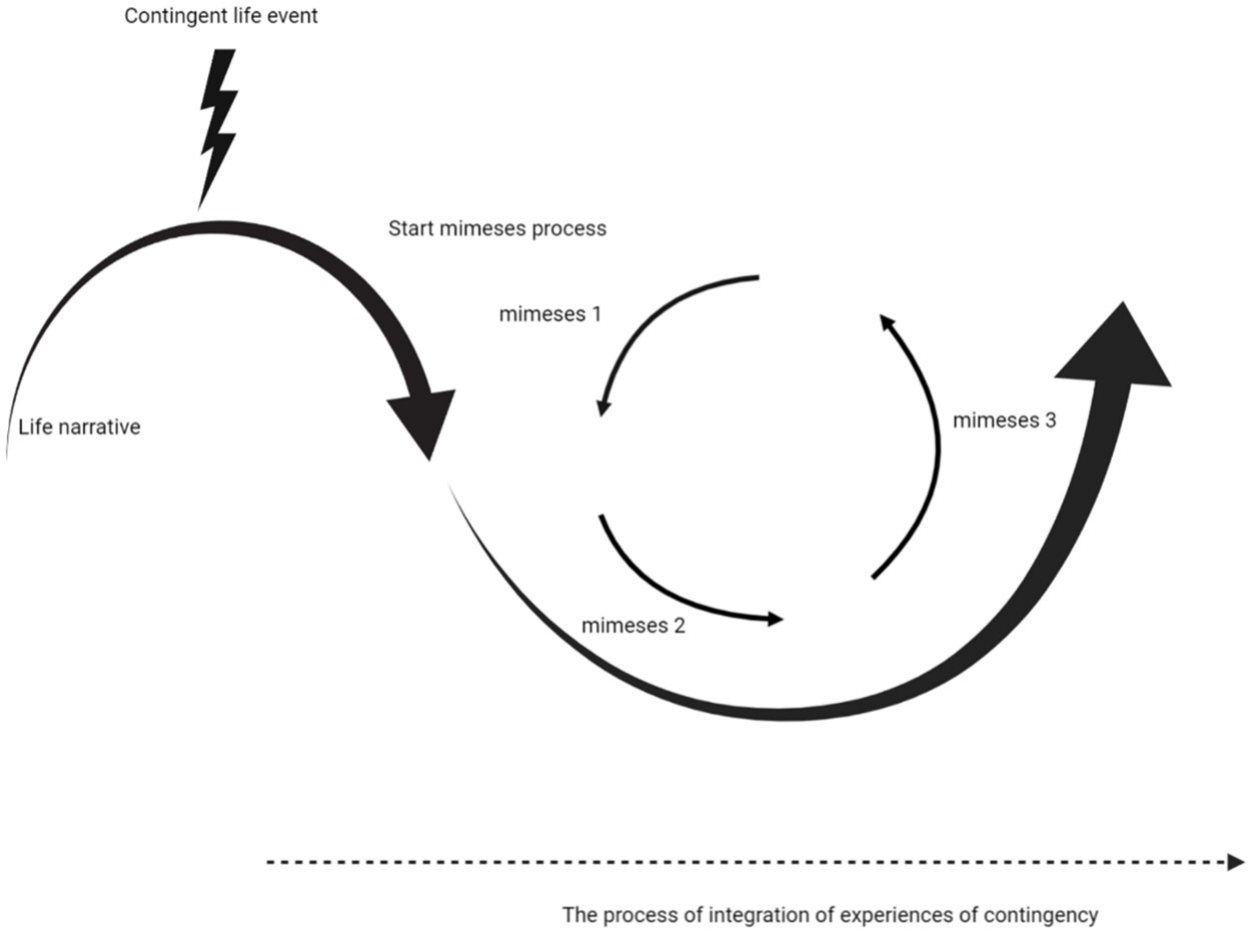
The relation between contingency, life narratives, mimesis and integration of experiences of contingency. After a contingent life event, which might result in an existential crisis, a process of integration of experiences of contingency starts to facilitate restoration of coherence and integration of this experience into the life narrative. The arrows indicate respectively a decrease and subsequent increase of coherence of the life narrative. This process has similarities with the mimesis process which describes the phases of development of a narrative concerning a specific experience, which as the cycle represents, is an iterative process where one can move through several mimesis cycles to process the experience of contingency until the experience feels sufficiently integrated into one’s life narrative.

A summary of the theoretical relation between contingency, life narratives, mimesis and integration of experiences of contingency is shown in Fig 1.

## 3. Methods

### 3.1 Participants

Professional artists participating in the In Search Of Stories project (ISOS) were interviewed [28]. ISOS evaluates a narrative multimodal intervention by professional artists and spiritual counsellors working with cancer patients aimed at enhancing quality of life [28]. The multi modal intervention included a life review interview, drawing of a Rich Picture [29], reading selected literature [28], and participating in a co-creation process. In total 11 artists were involved in ISOS, of which 10 were available for the interviews of the current study. Inclusion criteria included: experience with co-creative processes with patients, and being able to switch between their main art modality and other art modalities, thereby having a broader repertoire of working with patients. Exclusion criteria included: unavailability for the interviews for the current study. The purposive sample was intended to be gender balanced, including artists with experience in a variety of primary art forms as visual artists, musicians, scenographers, theatre makers and theatre writers. Table 1 illustrates the artist’s profession, age and gender. Their expertise with co-creation processes included working with patients in a hospital setting, community projects on traumatisation after the explosion of a firework factory, working with patients with epilepsy, social projects about poverty and trauma, working with children from deprived backgrounds and working with lonely elderly people. Artists were approached by telephone for one-on-one, in person interviews and didn’t have prior contact with the researchers. Artists were interviewed once. Interviews were conducted at the professional studio or at the home of the artist, according to their preference. Interviews took place between June 2020 and August 2020. The duration of the interviews was between 90 and 180 minutes, median duration was 120 minutes (two hours). As interviews were extensive no additional information was collected.

**Table 1:** Participating artists.

Field of expertise	Age	Gender
A: Musician^1^	42	Male
B: Musician	38	Male
C: Visual artist	64	Male
D: Visual artist	38	Female
E: Visual artist	53	Female
F: Theatre writer	61	Male
G: Scenographer	50	Female
H: Scenographer	49	Female
I: Theatre composer	50	Female
J: Creative writer	58	Female

^1^ A–J are used to reference the quotations in section 4. Results.

### 3.2 Study design, interviews and topic list

We used a qualitative research design and reported according to the COREQ Checklist [30], which is included in S1 Table. Data were collected through interviews, using a topic list and analysed with template analysis. The interviews were conducted by YW, who has a professional background in arts therapy, clinical psychology and spiritual care. All interviews were audio recorded for further analysis.

Based on currently available literature and discussions with the research group, we devised a topic list with distinct questions, which was used for the first two interviews and remained unchanged after these were analysed. The interview questions, which consisted of five subsections; ‘experience of contingency’, ‘life narratives’, ‘phases of mimesis’, ‘meaning’, and ‘artist-patient dynamics’, are shown in Table 2. Neutral follow-up questions were posed, such as, ‘Could you tell me more?’, ‘Can you please specify?’, to reveal additional information and explore deeper meaning [31]. The original Dutch version of the topic list is included in S2 Table.

**Table 2:** Topic list interview artists.

Topic Area	Questions
*1*. *Experience of contingency*	How can you use your expertise in art to recognise contingency? How can you label contingency for the patient? What is the aim of the co-creative process?
*2*.*Life narratives*	How is the patient’s life narrative being explored during the co-creation process? Where and how in the co-creative process is the emphasis on the life narrative of the patient, and on which aspects of the life narrative? How do you rework the life narrative of the patient?
*3*. *Phases of mimesis*	Are there distinct phases in the co-creation process? How does the co-creation process develop through time? When is the co-creative process successful to you?
*4*. *Meaning*	How do you, working within a co-creation process, recognise that patients contribute meaning to their life story? How do you perceive your artistic practice as a source of creativity? What can you say about the use and necessity of the senses in the co-creative process? What can you say about the use and necessity of symbolic language in the co-creative process?
*5*. *Artist*-*patient dynamics*	What is your standard approach towards the patient? What can you say about the relationship between artist and patient? How do you experience the level of alignment between the artist and the patient?

### 3.3 Data analysis

The audio recordings of the interviews were imported in AtlasTi [32] and analysed by means of template analysis [33, 34]. The main procedural steps of the analyses for the current study are illustrated in Table 3. The preliminary template, which was based on the topic list, and included five a priori themes, ‘experience of contingency’, ‘life narratives’, ‘phases of mimesis’, ‘meaning’ and ‘artist—patient dynamics’, was used to analyse the first two interviews, see Table 3 step 2 for details. This resulted in the second template which consisted of five themes, ‘expression of contingency’, ‘elements of co-creation’, ‘phases of mimesis’, ‘self-transcendence’ and ‘co-creation as a ritualizing process’, see Table 3 step 7 for details. Subsequently the final eight interviews were conducted and all 10 interviews were analysed, resulting in the final template with five themes, ‘expression of contingency’, ‘elements of co-creation’, ‘phases of co-creation’, ‘self-transcendence’ and ‘co-creation as a ritualizing process’, see Table 3 step 11 for details. The analyses were primarily conducted by YW (MA, MSc, MSc, female). Each phase, i.e., the a priori themes, the preliminary template and the second template, was discussed with MSR (Associate professor, PhD, male), who has a professional background in religious studies, theology, spiritual care, template analyses and qualitative research. The final template was discussed with MSR and HvL (Professor, MD, PhD, PhD, female), who has a professional background in oncology and theology. During analysis no difficulties in interpretation of the data occurred and subsequently no feedback on the transcripts was asked from the interviewed artists.

**Table 3:** Main procedural steps.

1. Based on literature and the topic list several a priori themes were identified by YW and discussed in detail with MSR. The a priori themes were initially labelled ‘experience of contingency’, ‘life narratives’, ‘phases of mimesis’, ‘meaning’ and ‘artist—patient dynamics’. 2. The a priori themes, ‘experience of contingency’, ‘life narratives’, ‘phases of mimesis’, ‘meaning’ and ‘artist—patient dynamics’, were included in the preliminary template used for data analyses. 3. By careful listening and extensive re-listening of the first two interviews YW familiarized herself with the interview data. 4. The preliminary template was used in the analyses of the first two interviews by YW. Themes and categories were distinguished in the imported data and labelled with a code. A priori themes were adapted or confirmed. New emerging themes were identified, defined and included. 5. YW organised the emerging themes into larger wholes of meaningful clusters and categories encompassing the preliminary understanding of the relationships between and within themes. 6. The results of this preliminary analysis were translated by YW into a second template which contained the tentative themes and codes of the first two interviews. These results were discussed with MSR. 7. In the second template the a priori themes ‘experience of contingency’ and ‘phases of mimesis’ were confirmed, albeit the theme ‘experience of contingency’ was renamed to ‘expression of contingency’. The a priori themes ‘life narratives’ and ‘artist—patient dynamics’ were combined into the theme ‘elements of co-creation’. The a priori theme ‘meaning’ was split into two themes, ‘self-transcendence’ and ‘co-creation as a ritualizing process’. 8. The final eight interviews were conducted by YW, according to the same topic list. 9. By careful listening and extensive re-listening of the interviews YW familiarized herself with the data of both the final eight interviews and the first two interviews. 10. Subsequently the full set of 10 interviews was analysed by YW in an iterative process where the template was continuously accommodated to contain all themes and codes. Themes and codes were modified to better represent the data. This iterative process continued until all interview data were mapped onto the template. Analysis indicated data saturation. 11. Finally, themes, codes and categories were grouped into concepts and constructs forming the final template which contained all themes and codes. The final template was discussed with MSR and HvL. In general the final template resembled and confirmed the second template, with the exception of ‘phases of mimesis’ which was replaced by ‘phases of co-creation’ as this offered a more specific representation of the data. The final themes were ‘expression of contingency’, ‘elements of co-creation’, ‘phases of co-creation’, ‘self-transcendence’ and ‘co-creation as a ritualizing process’. The theme ‘elements of co-creation’ was further specificized with six subthemes, see section 4. Results for details. The theme ‘phases of co-creation’ was further specificized with four subthemes of which subsequently in one a further two codes were identified, see section 4. Results for details.

### 3.4 Ethics

The study was exempted from ethical approval by the Medical Ethics Review Committee of the Academic Medical Centre, since the Medical Research Involving Human Subjects Act was not applicable (reference number: W20_436 # 20.483).

## 4. Results

### 4.1 Final template

**Fig 2:**
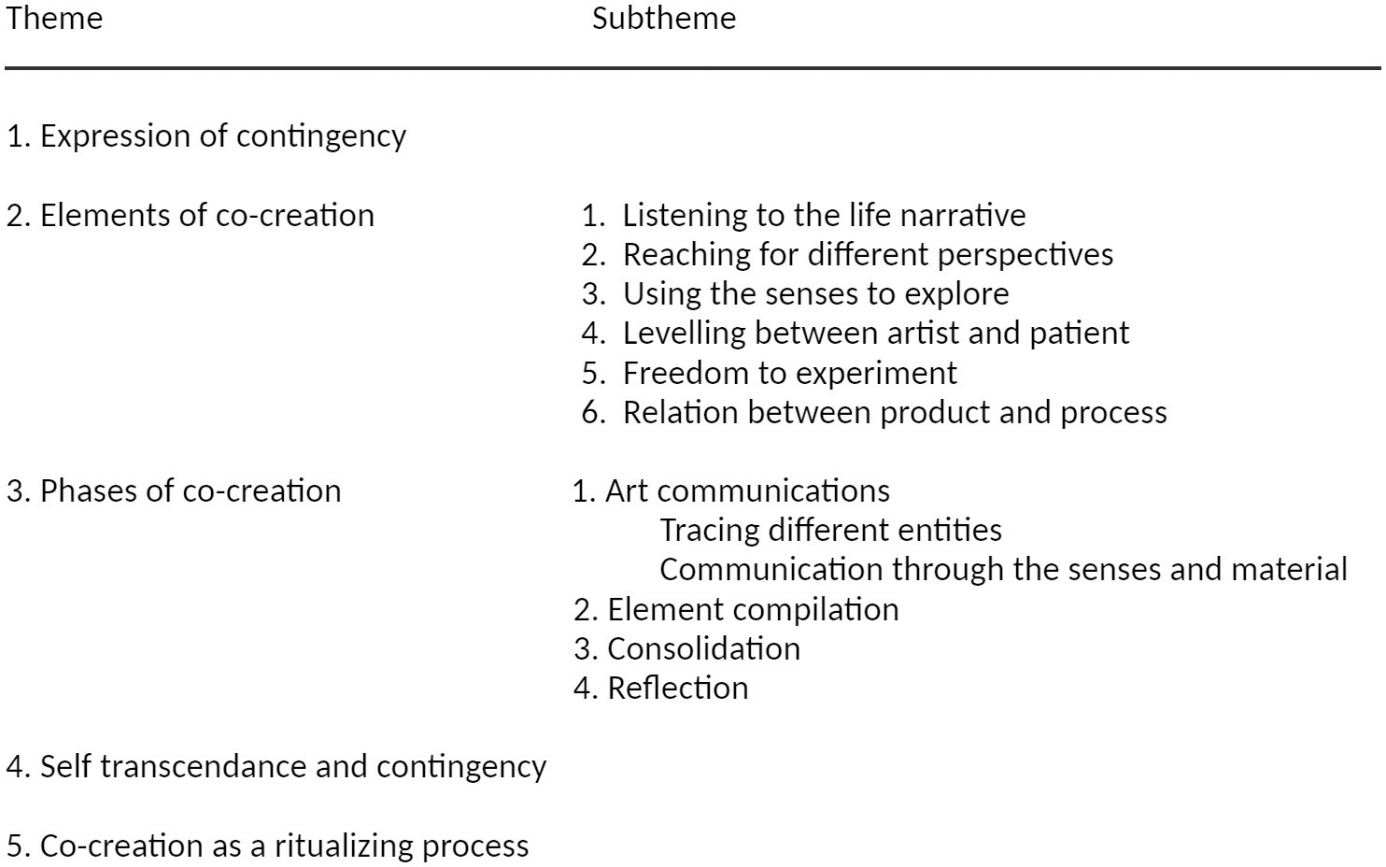
Final template. The subsequent phases of co-creation are numbered to indicate their temporal aspect.

The final template consisted of five themes: ‘expression of contingency’, ‘elements of co-creation’, ‘phases of co-creation’, ‘self-transcendence’, and ‘co-creation as a ritualizing process’. The themes ‘elements of co-creation’ and ‘phases of co-creation’ were further subdivided. The final template is shown in Fig 2.

### 4.2 Expression of contingency

The interviewed artists described it was important for their patients to give expression to (elements of) their life story and that patients also felt an urge to do so. The artists indicated to have a toolbox with ideas and art material handy to enable patients to express thoughts and emotions related to the experience of contingency. Artists elaborated on how art can be used to give expression to feelings and to reflect on these from different perspectives. Illustrative quotations reflecting artist’s thoughts on working with contingency are presented below.

*When facilitating training in creative writing*, *people mostly wanted to write down their life story*, *they weren*’*t much interested in the methodology of creative writing*, *perhaps because they wanted to report on the uniqueness of their life story*, *perhaps to explain things or leave something behind for others to reflect on*.

*(J)*

*A patient told me that he pictured that he was falling backwards in a pit and as he was falling he saw people disappear*. *He felt that only the shadows of people remained*. *To him this was a very strong image which we used for expression*.

*(H)*

*When we access a contingent life event I have my artistic toolkit to facilitate expression through words*, *feelings*, *associations or movement*, *but it is inevitable that we will use this toolkit to be able to tell the story from different perspectives*.

*(I)*

*To bear up de process of illness is an exhausting activity*. *Sometimes it can be so pleasant to be allowed to say*: “*I find it so heavy*, *so intense*”. *I suggest to patients to unravel that sense of heaviness or pain*. *Often they become very emotional*. *It can be a relief to name the process of mourning of things that are lost due to the illness*. *To not focus on surviving*, *accepting and appearing fine*, *but together focus on what actually happened to them*, *and how to put these feelings into a meaningful shape*.

*(D)*

### 4.3 Elements of co-creation

The final template indicated ‘elements of co-creation’ to consist of six subthemes, which are summarized below with their main characteristics and illustrative quotations.

#### 4.3.1 Listening to the life narrative

Artists described how they listen to the life narrative of the patient and ask questions to search for various ways in which patients both derive meaning from, and attach meaning to, their life narratives. The artists also reported they simultaneously looked for possibilities to translate this meaning into their own artistic language such as using different colours of paint or musical melodies.

*In the beginning of the process it is important to listen to the life narrative of the patients*, *what and how do they express themselves*, *what is their background and which choices did they make*? *What meaning does it have to them*, *and which aspects are of most importance*?

*Simultaneously I look for the auditive or musical realm that opens when they talk about these things and gradually*, *I introduce the language of music*.

*(A)*

#### 4.3.2 Reaching for different perspectives

The artists reported how they use the language of the art medium to create meaning and to create and recreate the life narrative from different perspectives, together with the patients.

*When I work with objects*, *we first have to create a language*, *then we answer the patients*’ *wishes concerning the meaning of the objects*. *Colour*, *textile*, *paper and music might play a role within creating meaning*. *Next we compose and recompose the story until it feels right*, *but the act of placing the objects is always meant to find a different perspective*.

*(G)*

#### 4.3.3 Using the senses to explore

The artists described how they support patients in their exploration through the use of the senses. For instance, supporting a patient to realise and become aware that touch is reciprocal: when the patient touches the material, he/she is also touched by the material.

*There is a reciprocity in touch when we work with materials*. *I touch something and I am touched by the material*. *Becoming aware that this happens invites the possibility to connect and receive information from the material*. *In my work I try to make that connection by working with for example silk*, *electricity wire and foam*.

*(H)*

#### 4.3.4 Levelling between artist and patient

The artists reported that they let the co-creation reveal itself instead of directing it. Also they follow the patient while they listen to, acknowledge and recognise the story of the patient. If requested by the patient, they give directions, otherwise, artist and patient discover the next step in close collaboration.

*The process reveals itself and is an interaction that revolves around listening*, *acknowledging and recognising*. *I follow them like a shadow instead of saying* ’*You have to go this way*’, *unless they ask for guidance*, *it is a constant checking which route we will take*.

*(J)*

#### 4.3.5 Freedom to experiment

Artists reported they facilitate freedom for patients to experiment by not directing the process and by keeping options open.

*Successful moments of co*-*creation are moments of very little words and much doing*, *being active in a space together*, *and after a while noticing that something has been created*. *There is no plan*, *no end result no linear roadmap*, *but improvising and searching together*. *I don*’*t like to exclude options*, *but rather follow impulses*.

*(B)*

#### 4.3.6 Relation between product and process

Artists described that the final artistic product only has value when the underlying process was meaningful.

*I don*’*t think we have to work towards an end product*, *the process is most important because that process will bring satisfaction to the composers*. *The process of turning an empty space into a mini theatre*. *The patient chooses an object and brings this into connection with other objects*. *What it eventually becomes is guided by the process*, *which reveals itself in the end*.

*(G)*

### 4.4 Phases of co-creation

The final template indicated ‘phases of co-creation’ to consist of four phases, which are summarized below with their main characteristics and illustrative quotations of artists.

#### 4.4.1 Phase 1: Art communications

Artists described the initial meeting with a patient as a process characterized by two further codes; tracing different aspects within the life story of the patient, and, communication through the senses and choice of art material within their specific expertise.

*As a musician I scan for the auditory space within the way the patient talks about an experience*. *It can be anything that is mentioned by the patient*.

*(A)*

*4*.*4*.*1*.*1 Tracing different aspects within the life story of the patient*. Artists reported they start co-creation with the exploration of the life story of the patient. They particularly look for important and different aspects of the patients’ life story. These sometimes opposing aspects of the life story are called the ‘different voices’ within the life story. During this exploration the artists also aim to uncover aspects the patient was not (fully) aware of.

*In the first phase the main goal is to trace different* ’*voices*’ *that are already present within the life narrative*. *The way a patient talks about this*, *what kind of meaning is attached to different aspects*. *Voices can be traced everywhere*: *in certain experiences*, *the surroundings of the patient*, *a certain life phase*, *people who are important to the patient*, *specific opinions etc*. *A woman with epilepsy talked about sounds she could hear that had a certain emotional meaning to her*. *We subsequently tried to make these* ’*voices*’ *concrete through sound frequencies and instruments*.

*(A)*

*4*.*4*.*1*.*2 Communication through the senses and choice of art material*. Artists indicated that they facilitate patients to use all the ‘senses’—touch, smell, hearing, vision, bodily sensations and emotions—as a first entry of exploring experiences of contingency within their life story. These experiences are further elaborated by focussing on certain elements in more detail through the use of one specific aspect of the senses. Subsequently this exploration is linked to specific art materials, which in turn are then used for a further expression of inner feelings.

*We looked for a mutual language derived from the senses*, *which is made abstract by creating something*. *We had to find a certain colour black*, *because that was part of his inner emotional experience*. *I have given him different pigments of black to make his own samples and to find the exact pigment for his feeling*.

*(C)*

#### 4.4.2 Phase 2: Element compilation

Artists further indicated that the elements originating from the expression of the ‘different voices’, as described in phase 1, are subsequently brought together in various combinations representing different story lines. These elements are iteratively combined into compilations until it feels right and fits a new perspective on one’s life story. Artists emphasized there are various possible outcomes for a compilation and the final outcome depends on the patient’s ‘choice’ which of the various life stories feels best.

*Each instrument has the ability to make a distinct tune which has a separate meaning to the patient*, *but together these tunes show a combined theme and rhythm*. *We can add and explore different rhythms and the height of any tonality until it feels right*. *The result can be several compositions or just one which fits everything in it*.

*(A)*

*I work with natural objects that are placed in a certain way to make a mini exposition on a table*. *We can use light and colour to emphasize certain elements and can replace the objects*, *which all have their own emotional value*, *to create a meaningful composition that reflects the story the patient wants to tell at that specific moment in time*. *This often requires concentration and focus*, *and at a certain point it is complete*.

*(G)*

*Practicing with voice tonality and text while presenting her life story in front of an audience helped her to organise her feelings*. *There was one sentence that she found really difficult to vocalise*. *Each time she articulated the words her voice would break and she would tear up*. *She had to become aware of her breathing and sense her body*, *and then try again*. *Eventually she could say the sentence with a sense of compassion towards herself*. *Working on that one sentence was transformational to her*. *This process simultaneously helped her to gain strength and discharge emotional load of her life story*.

*(I)*

#### 4.4.3 Phase 3: Consolidation

After the compilation, the actual work of art is created by the artist together with the patient or by the artist alone. The work of art is based on the elements that came forth from the expression and usually the art forms used earlier or comparable art forms are used for the design of the final artwork. The work of art is described as both a representation of the patient’s life story and of the process of co-creation.

*The work of art is a box in time where mental*, *emotional and spiritual aspects of the self are consolidated*, *it has its own memory capacity*. *It is like a heliographic print of the self*, *every act of composition is a distinct track within the work of art*.

*(C)*

#### 4.4.4 Phase 4: Reflection

The artists reported that they subsequently invite patients to reflect on the work of art, the new narrative and on the process of arriving there. The work of art has a capacity to stand on its own, transcending time, and every time it is observed the patient can allocate new meaning to it in one’s own unique way.

*The difference with the phase of consolidation is that when you are viewing the work of art*, *other emotions are revealed*. *You can be moved by it*, *you could see it as a treasure that has to be cherished*, *or perhaps a mirror that brings comfort*. *There is meaning in the fact that a patient is enabled to make that box in time and creates something that can be watched from a distance by him*-*/herself*.

*(C)*

### 4.5 Self-transcendence and contingency

Artists expressed that patients are able to move beyond the boundaries of their initial life story when they are able to endure an experience of contingency. This means that they can tolerate conflicting feelings and experiences, and accept that these do not have to be consistent, coherent or otherwise fit together.

*It is like a contingency* ’*contradiction*’: *What I mean is the ability to tolerate contrasting feelings*, *experiences and conclusions*. *That things don*’*t fit together and allow this to just be as it is*. *Because when someone is able to stay in that position*, *the possibility exists to move beyond the boundaries of the conditioned patterns the* ’*self*’ *has created*.

*(F)*

*By being able to be open to not*-*knowing*, *by being open to what is revealed*, *one is open to the unknown and therefore the whole experience is almost comparable to learning to bear the unknown*.

*(J)*

### 4.6 Co-creation as a ritualizing process

Some artists described the process of co-creation as having similar features to performing rituals, because in a ritual a specific set of proceedings leads to a climax which ultimately helps the performer to transcend beyond the boundaries of the self. They mentioned that in the art making process, once different elements are chosen for their importance and are being selected for compilation, every activity within the process is permeated with meaning, which could be described as ‘ritualized materialization’ because the process of meaning making has solidified in the work of art that was made. Also, the resulting work of art resembles the transactional objects of the ritualizing process, for example when a musical compilation reaches its point of height, or in a painting in which the patient sees reflections of the emotions encountered during the reworking of the life event.

*The process of selecting material and giving meaning to specific acts is supported by bringing thoughts*, *emotions and physical actions together into a significant focus*. *The accumulation of intensity of this focus is felt within the theatrical art making process*. *A group of patients working on a theatre performance about living in a rough area and under poor circumstances experienced an immense relief once the product was completed*, *as a climax had been reached and the group was released from the built up of energy within the production*. *The result was a highly potent and exultant atmosphere where all had transcended what they held as possible to reach*, *they had transcended their own expectations and thus had to welcome a new perspective about themselves and their own potential*.

*(I)*

*There is a sense of sacredness within every step of the art making process*, *one truly has to reach inside oneself to make multiple choices*, *each having a significant contribution to the whole process*. *In the end a monument is made which shows the transformational process of choices leading towards this specific painting*.

*(C)*

## 5. Discussion

Our research question was whether, and—if so—how, co-creation supports a process of integration of experiences of contingency, into the life narrative.

Our study showed that from the perspective of professional artists, co-creation supports a process of integration of experiences of contingency into one’s life narrative. Artists use the process of co-creation to support patients to rework an experience of contingency. The artists aim to facilitate patients in the processes of becoming aware of, and transcending, the current life narrative by use of all their senses. As such, co-creation can be seen as a specific approach to facilitate a process of integration of experiences of contingency, but is distinct from conversation or dialogue—which may also enable the process of integration of experiences of contingency—with respect to the use of (combinations of) all the senses, the use of art materials and the solidification of the co-creation process in a tangible work of art [25, 27].

The second part of our research question was how co-creation supports a process of integration of experiences of contingency into the life narrative. A caveat here is that, as we only interviewed artists, our findings need to be confirmed by patients themselves, which is the focus of future research. As described in our theoretical framework, the construct of mimesis includes both the process of the formation of one’s life narrative and the process of integration of experiences of contingency, leading to a constant (re)interpretation of one’s (narrative) identity [21, 22]. Mimesis describing the process of integration of experiences of contingency into one’s life narrative, could provide a useful entrance to deepen our understanding of how co-creation supports this process. Where mimesis can be viewed as a theoretical description, co-creation can be seen as a specific intervention supporting this process of integration of experiences of contingency into one’s life story. In co-creation an additional actor, i.e. the artist, is specifically involved in facilitating the process. The process of mimesis is solely based on the perspective of the person him- or herself.

Starting in phase one, ‘Art communications’, the artist puts a magnifying glass on the conscious and still unconscious, often opposing aspects of experiences of contingency in the life story of the patient by listening to the life narrative of the patient (Element 1). Artists use the senses and artforms (Element 3) to invite the patient to connect with aspects of their life story and there is an incentive for the patient to respond to this invitation. By doing so the patient becomes more aware of these aspects. Art communications thus seems to support the process of integration of experiences of contingency as theorized in mimesis 1, the prefiguration.

Subsequently, in phase 2, Element compilation, multiple storylines are explored and endowed with meaning (Element 2). Artists describe they follow an unconscious flow in which the co-creation process unfolds (Element 4) and that they facilitate freedom for patients to experiment with compilations of their life narrative (Element 5). These aspects support patients to come to a broader perspective on their life narrative. Thus, Element compilation seems to support the process of integration of experiences of contingency as theorised in mimesis 2, the configuration.

In co-creation phase 3, Consolidation, the new life narrative is also expressed in the visible form of a tangible work of art which shows resemblances to transactional objects. The work of art only has meaning when the process of arriving there has been meaningful for the patient (Element 6). As in mimesis no compilation of a work of art is included, this phase probably supports the process of integration of experiences of contingency, as theorised in mimesis, between mimesis 2, configuration, and mimesis 3, refiguration. Yet, the effect of such a tangible end product in the form of a work of art on the patient’s process of integration of experiences of contingency still needs to be clarified in future research.

Finally, in co-creation phase 4, Reflection, the artist invites the patient to reflect on all aspects encountered during the co-creation process. This could in itself lead to new perspectives on the life narrative. Within the co-creation process self-transcendence facilitates the patient moving towards more fluidity in the life narrative [16, 27]. Therefore, in this phase, self-transcendence forms the major factor influencing integration of experiences of contingency into one’s life narrative. Reflection seems to support the process of integration of experiences of contingency as theorised in mimesis 3, the refiguration. Still, the specific steps of how patients can reach this form of self-transcendence need to be defined.

**Fig 3:**
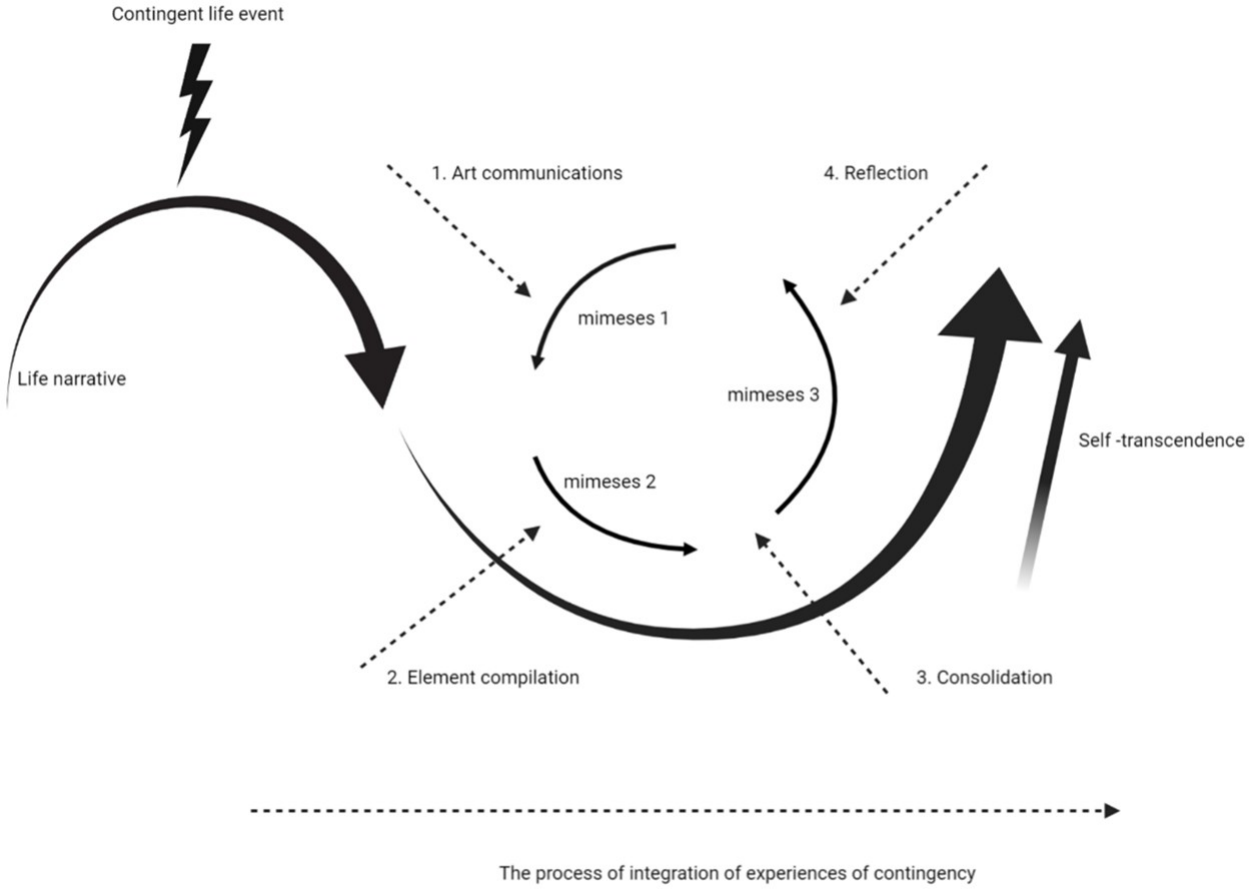
The position of co-creation within the theoretical framework. Co-creation consists of four distinct phases (Art communication, Element compilation, Consolidation, Reflection) which support certain aspects of the reworking and integration of a contingent life event into the life story, i.e. the integration of an experience of contingency as described in mimesis. The arrows indicate where the phases of co-creation affect the mimesis process, and thereby support the process of integration of experiences of contingency as described in the mimesis process. In both co-creation and mimesis, people can jump back and forth between phases, thus leading to an iterative process. Self-transcendence facilitates the patient to move towards more fluidity in the life narrative, enabling the integration of experiences of contingency.

Fig 3 shows an illustration of how co-creation supports the process of integration of experiences of contingency.

Although the use of the word ‘narrative’ in ‘life narrative’ puts an emphasis on the linguistic aspect of this concept, in this study it became clear that in the process of co-creation emphasis is placed on the non-linguistic, non-conversational aspects of integration of experiences of contingency. The interplay between the senses and the material facilitates an opening towards various and new ways in which patients experience aspects of their life narrative. Patients make contact with the material, which somehow resonates with their inner experience and subsequently are touched by this material.

It may be hypothesized that experiences through the senses—emotions and bodily sensations—in fact constitute the main components of one’s life narrative, while words are merely used to communicate this internally and externally. The search for interventions facilitating integration of experiences of contingency may therefore be broadened from linguistic based interventions to a wider field including interventions which exploit (combinations of all) the senses, which is currently investigated in the In Search Of Stories project [28].

Strengths of the study include interviews with professional artists who have extensive experience in various co-creative art projects and who have a high level of reflection. Also, the use of template analyses for the interpretation of the data helps to systematically organise and reflect on the data. A limitation of the study is that we do not know the viewpoint of patients regarding these processes, which could be different from the artists perspective. Also, in this study the artists were asked how the process in general evolves. More in depth analysis of the facilitators and barriers in the process of co-creation in relation to the integration of experiences of contingency would possibly provide useful information for practical applications. In future research we will observe these processes in a clinical setting as they evolve and interview patients and artists separately afterwards.

## 6. Conclusion

According to professional artists, co-creation can support the process of integration of contingent life events. As such, co-creation also supports the process of mimesis and adds the creation of a work of art constructed by artist and patient together, on which the patients can further reflect and find different perspectives. Self-transcendence, i.e. a change in one’s consciousness where one is pulled beyond the boundaries of one’s self, forms a pivot point for successful integration of experiences of contingency. Patients welcome new impulses to loosen rigid perspectives and move towards a more fluid life narrative which allows for new insights and experiences. In sum, co-creation, which focusses on experiences through the senses, could be of value to support patients with the integration of experiences of contingency into their life narratives.

## Supporting information

S1 Table COREQ checklist. COnsolidated criteria for REporting Qualitative research Checklist.
(PDF)

S2 Table Topic list. English and Dutch version.
(DOCX)
