# Supplementary material for: Co-creative art processes with patients: A theoretical framework and qualitative study among artists
Source: PLoS One. 2022 Apr 7;17(4):e0266401. doi: 10.1371/journal.pone.0266401 (PMC8989196; doi:10.1371/journal.pone.0266401)
Supplement: S2 Table — English and Dutch version. (DOCX) [file pone.0266401.s002.docx]

**S2 Table. Topic list.**

**English version**

1. Experience of contingency
 How can you use your expertise in art to recognise contingency?
 How can you label contingency for the patient?
 What is the aim of the co-creative process?

2.Life narratives
 How is the patient’s life narrative being explored during the co-creation process?
 Where and how in the co-creative process is the emphasis on the life narrative of the patient, and on which aspects of the life narrative?
 How do you rework the life narrative of the patient?

3. Phases of mimesis
 Are there distinct phases in the co-creation process?
 How does the co-creation process develop through time?
 When is the co-creative process successful to you?

4. Meaning
 How do you, working within a co-creation process, recognise that patients contribute meaning to their life story?
 How do you perceive your artistic practice as a source of creativity?
 What can you say about the use and necessity of the senses in the co-creative process?
 What can you say about the use and necessity of symbolic language in the co-creative process?

5. Artist-patient dynamics
 What is your standard approach towards the patient?
 What can you say about the relationship between artist and patient?
 How do you experience the level of alignment between the artist and the patient?

**Original Dutch version**

1 Ervaringen van contingentie
 Hoe kunt u uw expertise in het werken met kunst inzetten om contingentie te herkennen?
 Hoe kunt u contingentie benoemen voor de patiënt?
 Wat is het doel van het co-creatie proces?

2 Levensverhalen
 Hoe wordt het levensverhaal van de patiënt geëxploreerd tijdens het co-creatie proces?
 Waar en hoe ligt binnen het co-creatie proces de nadruk op het levensverhaal van de patiënt
 en op welke aspecten van het levensverhaal?
 Hoe bewerkt u het levensverhaal van de patiënt?

3 Fasen van mimesis
 Zijn er duidelijk te onderscheiden fasen binnen het co-creatie proces?
 Hoe ontwikkelt het co-creatie proces zich in de tijd?
 Wanneer is het co-creatie proces succesvol voor u?

4 Betekenis
 Op wat voor manier, werkend binnen een co-creatie proces, herkent u dat patiënten
 betekenis toekennen aan hun levensverhaal?
 Op wat voor manier ervaart u uw artistieke praktijk als een bron van creativiteit?
 Wat kunt u zeggen over het gebruik en de noodzaak van de zintuigen in het co-creatie
 proces?
 Wat kunt u zeggen over het gebruik en de noodzaak van symbolische taal in het co-creatie
 proces?

 5 Kunstenaar – patiënt dynamiek
 Wat is uw standaard benadering naar de patiënt?
 Wat kunt u zeggen over de relatie tussen de kunstenaar en de patiënt?
 Hoe ervaart u het niveau van uitlijning tussen de kunstenaar en de patiënt?
